# The Effect of Vaccine Type and SARS-CoV-2 Lineage on Commercial SARS-CoV-2 Serologic and Pseudotype Neutralization Assays in mRNA Vaccine Recipients

**DOI:** 10.1128/spectrum.00211-22

**Published:** 2022-03-21

**Authors:** Nicole V. Tolan, Amy C. Sherman, Guohai Zhou, Katherine G. Nabel, Michaël Desjardins, Stacy Melanson, Sanjat Kanjilal, Serina Moheed, John Kupelian, Richard M. Kaufman, Edward T. Ryan, Regina C. LaRocque, John A. Branda, Anand S. Dighe, Jonathan Abraham, Lindsey R. Baden, Richelle C. Charles, Sarah E. Turbett

**Affiliations:** a Department of Pathology, Brigham and Women’s Hospital, Boston, Massachusetts, USA; b Department of Pathology, Massachusetts General Hospital, Boston, Massachusetts, USA; c Department of Medicine, Division of Infectious Diseases, Brigham and Women’s Hospital, Boston, Massachusetts, USA; d Department of Medicine, Massachusetts General Hospital, Boston, Massachusetts, USA; e Harvard Medical School, Boston, Massachusetts, USA; f Department of Immunology and Infectious Diseases, Harvard T.H. Chan School of Public Health, Boston, Massachusetts, USA; Johns Hopkins Hospital

**Keywords:** mRNA vaccines, immunogenicity, serology

## Abstract

The use of anti-spike (S) serologic assays as surrogate measurements of SARS-CoV-2 vaccine induced immunity will be an important clinical and epidemiological tool. The characteristics of a commercially available anti-S antibody assay (Roche Elecsys anti-SARS-CoV-2 S) were evaluated in a cohort of vaccine recipients. Levels were correlated with pseudotype neutralizing antibodies (NAb) across SARS-CoV-2 variants. We recruited adults receiving a two-dose series of mRNA-1273 or BNT162b2 and collected serum at scheduled intervals up to 8 months post-first vaccination. Anti-S and NAb levels were measured, and correlation was evaluated by (i) vaccine type and (ii) SARS-CoV-2 variant (wild-type, Alpha, Beta, Gamma, and three constructs Day 146*, Day 152*, and RBM-2). Forty-six mRNA vaccine recipients were enrolled. mRNA-1273 vaccine recipients had higher peak anti-S and NAb levels compared with BNT162b2 (*P* < 0.001 for anti-S levels; *P* < 0.05 for NAb levels). When anti-S and NAb levels were compared, there was good correlation (all *r* values ≥ 0.85) in both BNT162b2 and mRNA-1273 vaccine recipients across all evaluated variants; however, these correlations were nonlinear in nature. Lower correlation was identified between anti-S and NAb for the Beta variant (*r *= 0.88) compared with the wild-type (WT) strain (*r *= 0.94). Finally, the degree of neutralizing activity at any given anti-S level was lower for each variant compared with that of the WT strain, (*P* < 0.001). Although the Roche anti-S assay correlates well with NAb levels, this association is affected by vaccine type and SARS-CoV-2 variant. These variables must be considered when interpreting anti-S levels.

**IMPORTANCE** We evaluated anti-spike antibody concentrations in healthy mRNA vaccinated individuals and compared these concentrations to values obtained from pseudotype neutralization assays targeting SARS-CoV-2 variants of concern to determine how well anti-spike antibodies correlate with neutralizing titers, which have been used as a marker of immunity from COVID-19 infection. We found high peak anti-spike concentrations in these individuals, with significantly higher levels seen in mRNA-1273 vaccine recipients. When we compared anti-spike and pseudotype neuralization titers, we identified good correlation; however, this correlation was affected by both vaccine type and variant, illustrating the difficulty of applying a “one size fits all” approach to anti-spike result interpretation. Our results support CDC recommendations to discourage anti-spike antibody testing to assess for immunity after vaccination and cautions providers in their interpretations of these results as a surrogate of protection in COVID-vaccinated individuals.

## INTRODUCTION

Since the emergence of the COVID-19 pandemic, SARS-CoV-2 serologic assays have been an important component of the SARS-CoV-2 response, with over 90 assays receiving emergency use authorization (EUA) from the Food and Drug Administration (FDA). Many of these assays detect antibodies targeting the viral spike (S) protein in persons with past SARS-CoV-2 infection and those who have received COVID-19 vaccines (1). As more people become immunized with COVID-19 vaccines, understanding vaccine-induced antibody responses will be important to address new questions that arise with the dynamics of the pandemic, such as protection against SARS-CoV-2 variants. Additionally, although the Centers for Disease Control and Prevention (CDC) does not currently recommend serologic testing for COVID-19 vaccination response (2), this may change in the future as the correlation between antibody levels and protective immunity is further elucidated (3). High throughput, commercially available serologic assays could be essential to help determine protection against SARS-CoV-2 variants on a population-level, and the immunogenicity outputs will potentially help inform public-health decisions regarding additional booster vaccinations and/or novel SARS-CoV-2 vaccine development.

The U.S. FDA authorized two mRNA SARS-CoV-2 vaccines for emergency use in December 2020: mRNA-1273 (ModernaTX, Inc) and BNT162b2 (Pfizer-BioNTech), with BNT162b2 receiving full approval for individuals ≥ 16 years old and EUA for children ≥5 years old in August and October 2021, respectively (4–6). Both vaccines are administered as two-dose series, 21 (BNT162b2) or 28 days (mRNA-1273) apart, demonstrated 94% to 95% efficacy in clinical trials (7, 8), and have since received EUA for a single booster ≥ 6 months after the primary series (9). These mRNA vaccines elicit antibodies against the viral spike (S) protein that block S protein receptor-binding domain (RBD) interactions with the angiotensin-converting enzyme 2 (ACE2) receptor or prevent the S protein from mediating viral-host membrane fusion, thus stopping host cell infection (10, 11).

SARS-CoV-2 S protein neutralizing antibody (NAb) levels have been shown to be predictive of immune protection against symptomatic infection and have correlated with protective immunity against SARS-CoV-2 in human and animal studies (12–14). While NAbs may be helpful to measure vaccine-induced protection, assays measuring NAbs are predominantly available in the research setting only, limiting their clinical utility. Numerous studies have revealed strong association between NAb and anti-S levels in previously SARS-CoV-2 infected subjects (15–17), suggesting that SARS-CoV-2 serologic assays targeting the S protein could serve as practical surrogates for neutralizing activity. Thus, determining the degree of this correlation in COVID-19 vaccinated individuals and understanding variables that affect the correlation would be useful to predict viral neutralizing activity.

In this study, we measured anti-S total immunoglobulin (Ig) using the commercially available Roche SARS-CoV-2 anti-S assay (Elecsys anti-SARS-CoV-2 S antibody assay, Roche Diagnostics, Indianapolis, IN) in a cohort of immunocompetent individuals who received mRNA-1273 or BNT162b2 vaccination. We aimed to (i) assess the longitudinal kinetics of anti-S titers as measured by the Roche Anti-S SARS-CoV-2 S assay, (ii) compare total Ig and isotype antibody responses between mRNA-1273 and BNT162b2 vaccine recipients, (iii) compare NAb levels between mRNA-1273 and BNT162b2 vaccine recipients across SARS-CoV-2 variants using pseudotype neutralization (PN) assays, (iv) correlate the Roche anti-S antibody response with NAb levels, and (v) compare the correlation between anti-S and NAb levels by vaccine type and SARS-CoV-2 variant. In addition to evaluating circulating variants in the community (wild-type, Alpha, Beta, and Gamma), we also evaluated three constructs, Day 146*, Day 152*, and RBM-2, derived from a single immunocompromised patient infected with SARS-CoV-2 who developed mutations in the RBD over time (18) to further evaluate how viral evolution and mutations affect the anti-S and NAb relationship.

## RESULTS

### Participant demographics.

A total of 50 participants were enrolled in the study; one was excluded due to a history of COVID-19, one was excluded due to a history of immunosuppression, and two were lost to follow-up, with 46 participants included in the final analysis. The median age of the participants was 28 years (IQR 23.2 to 51.0); 28 (60.9%) were female and 34 (73.9%) were white (Table 1). Of the 46 total participants included, 28 (60.9%) received the mRNA-1273 vaccine and 18 (39.1%) received the BNT162b2 vaccine; all participants completed the two-dose series. All participant samples included in the analysis were negative for anti-N antibodies at baseline and at each time point of sample collection.

**TABLE 1:** Vaccine recipient demographics

Demographic	All participants (*n* = 46)	mRNA-1273 (*n* = 28)	BNT162b2 (*n* = 18)
Median age (IQR)	28 (23.2–51.0)	24.8 (23.5–51.1)	38.6 (23.8–58.5)
Female sex, no. (%)	28 (60.9)	16 (57.1)	12 (66.7)
Race, no. (%)			
White	34 (73.9)	22 (78.6)	12 (66.7)
Black	5 (10.9)	2 (7.1)	3 (16.7)
Asian	5 (10.9)	3 (10.7)	2 (11.1)
Native American/Alaskan Native	1 (2.2)	0 (0)	1 (5.6)
Other	1 (2.2)	1 (3.6)	0

### Convalescent plasma donor demographics.

**FIG 1:**
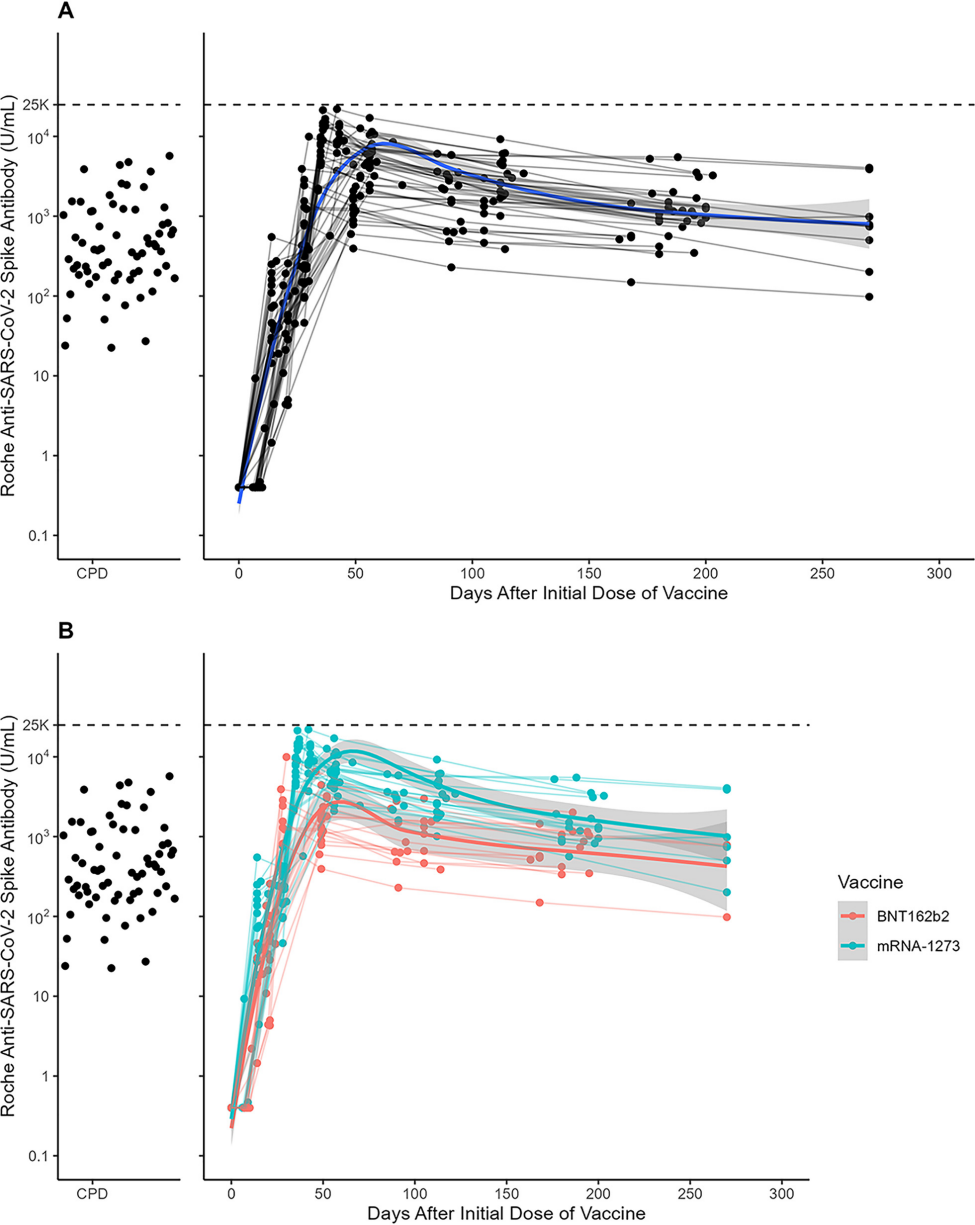
Roche anti-SARS-CoV-2 spike total antibody levels (U/mL) in (A) all mRNA vaccine participants (N = 46) and (B) mRNA-1273 (red, *n* = 28) and BNT162b2 (blue, *n* = 18) vaccine participants following vaccination. Each dot represents a unique measurement of spike total antibody levels; best-fit lines (Loess fit) are shown and colored according to vaccine type with corresponding 95% CI represented in gray. Individual Roche anti-SARS-CoV-2 spike total antibody levels measured in sera and/or plasma from convalescent plasma donors (CPD, *n* = 66) are represented by the black dots on the left for comparison. The black dashed line indicates the upper limit of quantitation for the Roche anti-SARS-CoV-2 spike total antibody assay.

Samples from 66 participants eligible for convalescent plasma donation were included in the analysis. Convalescent plasma donation (CPD) demographics are described in Table S1. Median anti-S levels measured from CPD individuals was 377.6 U/mL (IQR 188.4 to 1116.8) (Fig. 1A).

### Anti-S response in vaccine recipients.

At baseline, all participants had negative (<0.80 U/mL) anti-S antibody results. Anti-S concentrations began to increase after the first mRNA vaccine dose with 46/46 (100%) participants having detectable anti-S levels 14 days after their first vaccination. After vaccine dose 2, there was a sharp rise in anti-S levels with concentrations peaking approximately 49 to 56 days after the first vaccine dose (28 days post-vaccine dose 2) (median anti-S 4382.5, IQR 1787.5 to 6640.0; Fig. 1A). Anti-S antibody levels then began to decrease over time with a median anti-S level of 767.5 (IQR 743.5 to 932.5) 263 to 270 days after the first vaccine dose. Median anti-S levels were higher in samples from vaccinated participants compared to those from CPDs at all time points after day 49 (all time points *P* < 0.001, Fig. 1).

### Comparison of BNT162b2 and mRNA-1273 total anti-S and isotype antibody levels.

Approximately 49 to 56 days after vaccine dose 1, anti-S levels peaked and were significantly higher in individuals who received mRNA-1273 vaccination (median anti-S: 6358.5) compared with those who received BNT162b2 (median anti-S: 1469.5) (*P* < 0.001, Fig. 1B). Antibody isotype analysis from a subset of these samples (*n *= 20) at peak antibody levels (49 to 56 days post-vaccine dose 1) revealed higher concentrations of IgA (median anti-RBD IgA: mRNA-1273, 2.1; BNT162b2, 0.6; *P* = 0.02) and trend toward higher IgG anti-RBD levels (median anti-RBD IgG: mRNA-1273, 15.0; BNT162b2, 9.3; *P* = 0.08). IgM anti-RBD levels were similar between the two vaccine types (median anti-RBD IgM; mRNA-1273, 0.8; BNT162b2, 0.4; *P* = 0.02 Fig. S1).

### Comparison of neutralizing antibody levels by vaccine type and SARS-CoV-2 variant.

**FIG 2:**
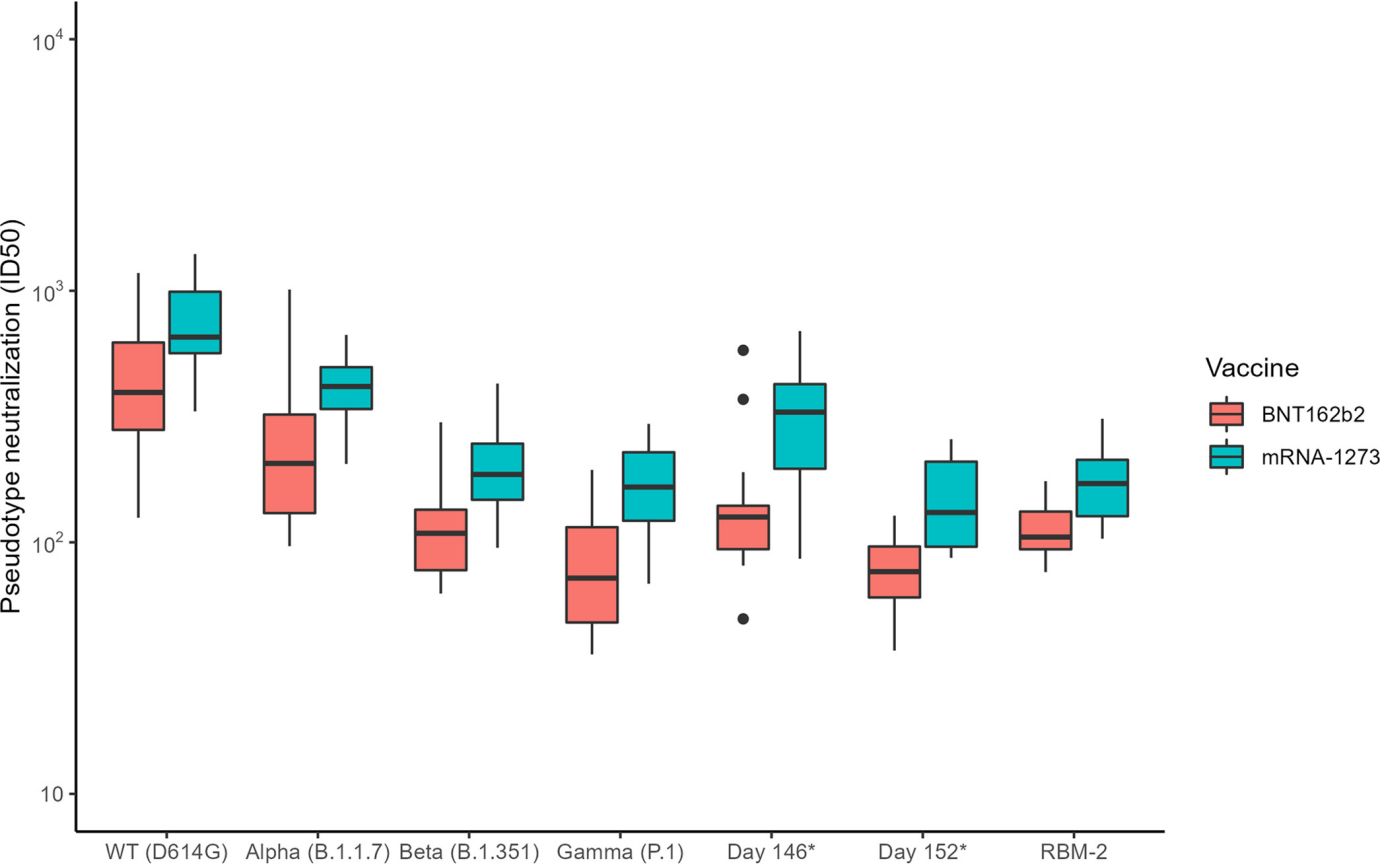
Comparison of pseudotype neutralization (PN, measured by ID_50_) levels between mRNA-1273 (*n* = 28) and BNT162b2 (*n* = 18) vaccine recipients, collected 49 to 56 days after the first vaccine dose (28 days after the second vaccine dose) across all evaluated variants. For each variant, there was a significant difference between PN levels from mRNA-1273 and BNT162b2 vaccine recipients (*P* < 0.05). Black horizontal bars denote median values and the vertical bars denote interquartile range (IQR). Day 146*, day 152*, and RBM-2 are constructs that contain composite spike protein mutations to the N terminal domain and RBD, derived from an immunocompromised individual with persistent infection.

PN was performed on serum from mRNA vaccine recipients (*n* = 46) at the sample collection times described above using the WT (defined as pseudotype containing the D614G S mutation) (19), Alpha, Beta, and Gamma pseudotypes as well as three additional pseudotypes with further spike protein mutations to the N terminal domain and RBD (day 146*, day 152*, and RBM-2) (Table 2). PN was performed on sera from 119 time points for WT, 116 time points for Alpha, 113 for Beta, 112 for Gamma, 104 for day 146*, 97 for day 152*, and 59 for RBM-2 pseudotypes. Overall, peak NAb levels (49 to 56 days post dose 1, measured by ID_50_) were lower for all evaluated variants compared to those measured for the WT strain in both mRNA-1273 and BNT162b2 vaccine recipients (*P* < 0.01) (Fig. 2, Table S2). Additionally, median NAb levels were significantly lower in BNT162b2 vaccine recipients compared to those who received mRNA-1273 for all SARS-CoV-2 strains, including WT (all values *P* < 0.05) (Fig. 2, Table S2).

**TABLE 2:** Key mutations present in the SARS-CoV-2 variant tested in pseudotype neutralization assays on sera from mRNA vaccine recipients^*a*^

Key mutations	SARS-CoV-2 variant						
	WT (D614G)	Alpha (B.1.1.7)	Beta (B.1.351)	Gamma (P.1)	Day 146*	Day 152*	RBM-2
D614G	+	+	+	+	+	+	+
K417 N/T			+	+			+
N440D					+		
T478K					+	+	+
E484K/A			+	+		+	+
F486I						+	
Y489H					+	+	+
S494P					+	+	+
Q493K					+		+
N501Y		+	+	+	+	+	+

a WT, wild type. + sign, mutation present.

### Correlation between anti-S level and SARS-CoV-2 pseudotype neutralization.

**FIG 3:**
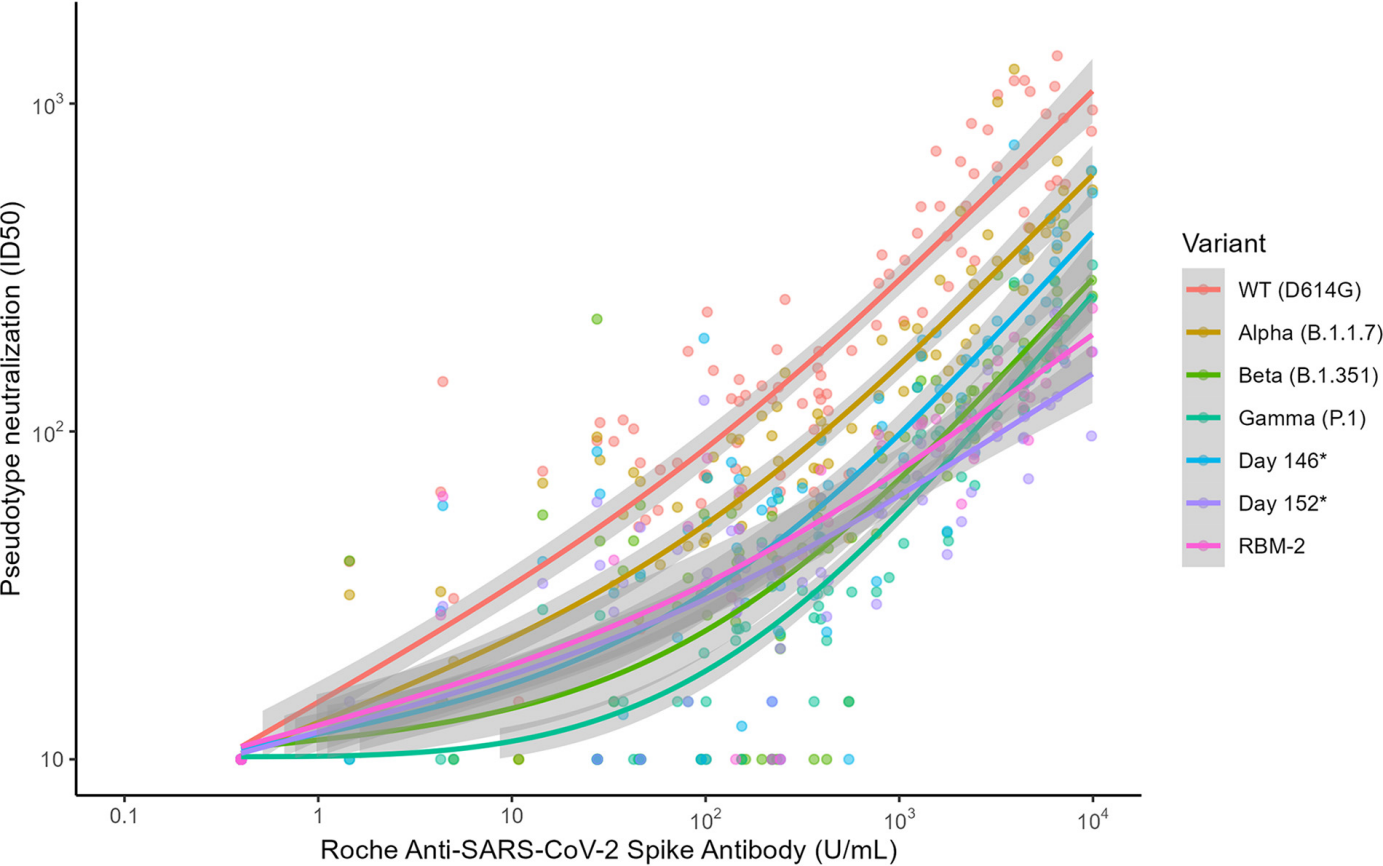
Pseudotype neutralization (ID_50_) titers as a function of anti-S levels (U/mL) colored by each SARS-CoV-2 strain. Best-fit lines (GAM fit) are shown with corresponding 95% CI represented in gray. ID_50_, 50% inhibitory dilution. Day 146*, day 152*, and RBM-2 are constructs that contain composite spike protein mutations to the N terminal domain and RBD, derived from an immunocompromised individual with persistent infection.

**FIG 4:**
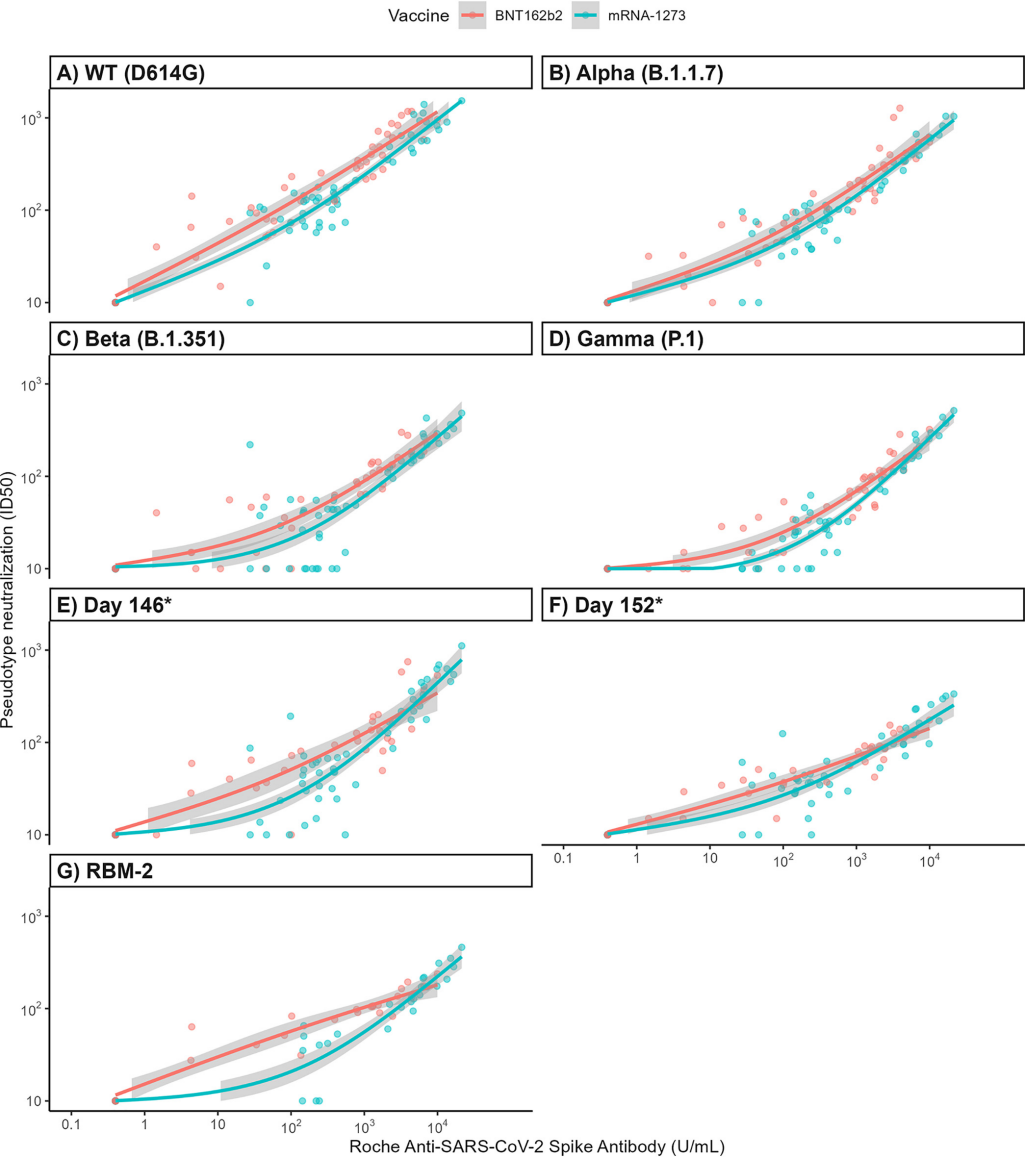
Pseudotype neutralization (ID_50_) titers as a function of anti-S levels (U/mL) colored by vaccine type and separately plotted for each SARS-CoV-2 strain (panels A to G). Best-fit lines (GAM fit) are shown with corresponding 95% CI represented in gray. ID_50_, 50% inhibitory dilution; WT, wild type. Day 146*, day 152*, and RBM-2 are constructs that contain composite spike protein mutations to the N terminal domain and RBD, derived from an immunocompromised individual with persistent infection.

To determine if the correlation between anti-S levels and PN is affected by vaccine type or SARS-CoV-2 variant, we first correlated anti-S levels with PN for the WT strain. Overall, anti-S levels correlated well with NAb levels when samples from all vaccine recipients were analyzed together (*r *= 0.94) (Table 3) or independently (*r *= 0.95 for BNT162b2; *r *= 0.93 for mRNA-1273); however, this relationship was non-linear (*p* < 0.01) (Fig. 3, Fig. 4A).

**TABLE 3:** Spearman correlation coefficients from regression analyses of pseudotype neutralization (ID_50_) as a function of anti-S levels (U/mL) in sera from mRNA vaccinated individuals

Correlation coefficient (*r*), (95% CI)^*a*^							
Vaccine type	Variants						
	WT^*b*^	Alpha	Beta	Gamma	Day146*	Day152*	RBM-2
BNT162b2	0.95 (0.91 to 0.98)	0.96 (0.92 to 0.98)	0.93 (0.86 to 0.97)	0.95 (0.89 to 0.98)	0.89 (0.78 to 0.95)	0.95 (0.89 to 0.98)	0.93 (0.83 to 0.98)
mRNA-1273	0.93 (0.88 to 0.96)	0.93 (0.89 to 0.96)	0.85 (0.75 to 0.91)	0.92 (0.86 to 0.95)	0.90 (0.83 to 0.94)	0.89 (0.80 to 0.94)	0.96 (0.91 to 0.98)
Combined	0.94 (0.91 to 0.96)	0.95 (0.93 to 0.97)	0.88 (0.82 to 0.92)	0.93 (0.90 to 0.96)	0.91 (0.86 to 0.94)	0.91 (0.87 to 0.95)	0.94 (0.90 to 0.97)

a Correlations are represented for each SARS-CoV-2 variant lineage and mRNA vaccine type (BNT162b2, mRNA-1273, and combined). Day 146*, day 152*, and RBM-2 are constructs that contain composite spike protein mutations to the N terminal domain and RBD, derived from an immunocompromised individual with persistent infection.

b WT, wild type.

We next correlated anti-S levels with PN for SARS-CoV-2 variants being monitored (Alpha, Beta, Gamma) as well as three additional variant pseudotypes with additional spike protein mutations (day 146*, day 152*, RBM-2) in 46 vaccine recipients (Fig. 3), and then further delineated the correlations by vaccine type and variant (Fig. 4A to G). Overall, there was a positive correlation between anti-S and NAb levels across all evaluated variants for both vaccine types (all r values ≥ 0.88) (Table 3), but again, these correlations were non-linear (*P* < 0.01 for all variants). Furthermore, the degree of correlation was not uniform across all variants, with a trend toward a lower correlation for the Beta variant (*r *= 0.88, 95% CI = 0.82 to 0.92) compared with the WT strain (*r *= 0.94, 95% CI = 0.91 to 0.96). Additionally, due to decreased NAb levels by SARS-CoV-2 variants compared with WT, the degree of neutralizing activity at any given anti-S level was lower for each variant compared with that of the WT strain, with more pronounced effects seen in Beta, Gamma, day 152*, and RBM-2 variants (*P* < 0.001 for all variants across both vaccine types combined) (Fig. 3). When analyzed by vaccine type and strain, the neutralizing activity to anti-S level association was also significantly different for each variant compared to the WT strain, except for the Alpha variant (*P* = 0.0517) for BNT162b2 vaccine recipients.

## DISCUSSION

As COVID-19 vaccination efforts continue across the globe, an increasing percentage of the population is now immunized against SARS-CoV-2. Vaccine-induced immunity for both individual assessment and population surveillance will be important to further understand protection against variants and to determine guidelines for additional vaccine doses and optimal timing. Although NAb levels are currently recognized as the best estimation of protection against COVID-19 (13), neutralization assays are costly, labor-intensive, and require specialized laboratory equipment and techniques for performance, making them impractical for widespread clinical use. In comparison, high-throughput, low-cost, commercially available anti-S antibody tests, such as the Roche Elecsys anti-SARS-CoV-2 S assay, have already been widely implemented in numerous clinical settings across the country.

In this study, we describe the kinetics of the Roche anti-SARS-CoV-2 S assay in immunocompetent, SARS-CoV-2 mRNA vaccine recipients up to 8 months post-initial vaccination and illustrate the correlation between anti-S and NAb levels against WT and variant SARS-CoV-2 strains. Similar to prior studies, we demonstrate that anti-S levels peak 49 to 56 days after the first vaccine dose, with a durable but decreasing response up to 8 months after initial vaccination in both mRNA-1273 and BNT162b2 vaccine recipients (20, 21). Median anti-S levels 49 days and beyond first dose were higher than those from high-titer CPD individuals, who were a median of 70 days from a positive SARS-CoV-2 PCR test. In addition, higher peak anti-S antibody titers were found in mRNA-1273 vaccine recipients compared to those who received BNT162b2 during the study period, which is consistent with previously reported data (22).

We also demonstrated differences in anti-SARS-CoV-2 S protein immunoglobulin isotypes and peak NAb levels in sera from vaccinated individuals according to vaccine type received. Participants who received mRNA-1273 had higher levels of anti-S IgA and IgG antibodies as well as higher peak NAb levels, compared with those who received BNT162b2. These differences may be explained by the higher mRNA content present in each dose of the mRNA-1273 vaccine (100 μg versus 30 μg in BNT162b2), and the subsequent larger concentration of antigen produced per dose. The different formulations of the lipid nanoparticle coat of mRNA-1273 and BNT162b2 may also be responsible for the different levels of the isotypes induced, with the hypothesis that the lipid components of the mRNA vaccines may increase Th1-polarized CD4^+^ T-cell responses and lead to early IgG class- switching with higher IgG levels compared with IgA and IgM (23). Overall low IgM and IgA responses to mRNA vaccines have been described (especially compared with responses post-natural infection) which is consistent with our findings (24). The greater breadth of isotype and NAb response from mRNA-1273 compared with BNT162b2 vaccinated recipients is unlikely to correlate with increased protection, at least for WT strains in the first month post-vaccination, because efficacy trials for mRNA-1273 and BNT162b2 demonstrated similar results. However, data from a real-world evaluation demonstrated that vaccine effectiveness for BNT162b2 begins to decline 5 months after vaccination (20), while vaccine efficacy as described by the phase 3 trial for mRNA-1273 remained high at 5 months after vaccination (25). These findings suggest that the differences in the magnitude of NAb levels and possibly the specific antibody isotype response induced by the mRNA-1273 vaccine series may be beneficial for the durability of protection. Because only a subset of the participant samples were evaluated for immunoglobulin isotypes, further studies are warranted to confirm these findings.

When anti-S and NAb levels were compared, nonlinear relationships were described for all vaccine types and variants, with good overall correlation. However, although all anti-S and PN were well correlated for each strain independently, the relationship between anti-S and PN was unique according to the individual variant being tested with the degree of neutralizing activity at any given anti-S level dependent on vaccine type received and SARS-CoV-2 variant. Additionally, some variants (Beta) showed lower correlation between anti-S and NAb levels compared with the WT strain. Notably, the SARS-CoV-2 constructs (day 146*, day 152* and RBM-2), containing composite mutations originally derived from a persistently infected immunocompromised host (18), were more resistant to neutralization by sera from vaccinated recipients. Because the Omicron variant shares many of the same key mutations as RBM-2 in the RBD (e.g., at positions K417, T478, E484, Q493, and N501), similar effects on neutralizing activity are predicted. These results demonstrate that many factors affect the relationship between Roche anti-S and NAb levels, complicating the interpretation of these values as a surrogate marker of immunity, particularly as variants containing larger numbers of RBD substitutions emerge.

Our data are limited by the relatively small sample size. In our comparisons between mRNA-1273 and BNT162b2 recipients, we did not match by age or gender; variables which may impact antibody magnitude and kinetics over time. The timing of anti-S level measurement in relation to vaccine administration likely affects the correlation between anti-S and NAb levels, due to differential induction and decay of anti-S and PN antibodies over time (21); however, our study was not powered to detect differences for this variable. Finally, our study did not evaluate clinical efficacy directly, and thus the degree of protection provided by specific anti-S and neutralization thresholds is not known.

In conclusion, although the Roche anti-S assay does generally correlate well with SARS-CoV-2 neutralizing antibodies, this correlation is nonlinear in nature and the strength of the association between these variables differs according to vaccine type and specific variant strain. In addition, the degree of neutralizing activity at any given anti-S level is variable and dependent on both vaccine type and SARS-CoV-2 variant, further complicating the interpretation of these values as a surrogate marker of immunity. These findings are particularly significant as new variants such as Omicron emerge; laboratory interpretations and methods must not be static and need to adapt as SARS-CoV-2 continues to evolve. By applying a “one size fits all” approach to interpretation, clinicians risk overestimating the potential immune correlates associated with anti-S levels, which could lead to incorrect guidance regarding an individual’s COVID-19 infection risk and need for mitigation strategies. Given all these factors, the use of anti-S levels to inform decisions regarding SARS-CoV-2 immunity should be avoided and the above nuances must be taken into consideration when making decisions regarding testing and reporting in the clinical setting.

## MATERIALS AND METHODS

### Participant samples.

A prospective study was conducted at Brigham and Women’s Hospital (BWH) and Massachusetts General Hospital (MGH) from December 2020 to July 2021 and approved by the Mass General Brigham Health System (2020P001580, 2020P000849, 2014P002602, 2006P001197) Institutional Review Board. Adults (≥18 years old) who were planning to receive a two-dose series of BNT162b2 or mRNA-1273 were recruited. Written informed consent was obtained from all participants. Participants were excluded if they had any of the following: immunosuppressive conditions, asplenia, HIV with CD4 count <200 and/or detectable HIV viral load in the last year, receipt of systemic immunosuppressive or immune-modifying therapies for ≥14 days within 6 months of enrollment, or pregnancy. For each participant, venous blood samples were collected prior to vaccination (day 0); 7 or 14 days after the first dose; at the time of the second dose (approximately 21 or 28 days depending on vaccine administered); and after the second dose, at approximately 49 to 56, 84 to 91, 105 to 112, 175 to 197, and 263 to 270 days post-first dose. Longitudinal samples collected after a booster dose were excluded from analysis.

### Convalescent plasma donors.

To compare the participants' anti-S levels to those induced by natural SARS-CoV-2 infection, sera from individuals with a history of PCR-confirmed COVID-19 infection who were eligible for potential CPD were obtained from the Mass General Brigham Biobank. Each sample came from an individual with a history of COVID-19 within the last 6 months. Those who previously received convalescent plasma, SARS-CoV-2-specific monoclonal antibodies, or SARS-CoV-2 vaccination were excluded. Each CPD sample had SARS-CoV-2 NAb testing performed with ID_50_ >100, according to the EUA guidelines for convalescent plasma (26).

### Serologic assays.

**(i) Roche Elecsys anti-SARS-CoV-2 S and anti-SARS-CoV-2 assays.** To measure anti-S antibody levels in vaccine recipients, all samples were analyzed using the Roche Elecsys anti-SARS-CoV-2 S assay, which is an electrochemiluminescence immunoassay for the qualitative and semi-quantitative detection of IgM, IgA, and IgG antibodies to the SARS-CoV-2 S protein RBD in human plasma or serum (27). Reported sensitivity and specificity are 93% to 98% and 99.9% to 100% (95% CI = 99.9% to 100%) in individuals with PCR confirmed COVID-19 infection (1, 28). The positive threshold is a cutoff index (COI) of ≥0.8 U/mL; the lower and upper limits of quantitation are 0.4 U/mL and 25,000 U/mL, respectively. Linearity studies using commercial materials (CalCheck Anti-SARS-CoV-2 S, Roche Diagnostics, Indianapolis, IN) and manual dilution studies of subject samples were conducted to confirm the COI and the upper limit of the reportable range (data not shown). Plasma and serum samples were analyzed on the cobas e601 as described in the package insert.

To determine subclinical SARS-CoV-2 infection during the study period, all samples were concomitantly analyzed on the anti-nucleocapsid (N) Roche Elecsys anti-SARS-CoV-2 assay (Roche Diagnostics, Indianapolis, IN). Plasma samples were analyzed as described in the package insert.

**(ii) Anti-SARS-CoV-2 isotype specific enzyme-linked immunosorbent assay.** To identify and correlate isotype specific responses to the RBD protein in mRNA vaccinate recipients with the anti-S assay, plasma IgM, IgA, and IgG isotype levels were measured using a laboratory developed enzyme-linked immunosorbent assay (ELISA), as previously described (16).

**(iii) Neutralization assays.** To determine NAb levels in mRNA vaccinated individuals, lentivirus pseudotypes for D614G (WT), and variants Alpha (B.1.1.7), Beta (B.1.351), and Gamma (P.1) were produced. In addition, lentivirus pseudotypes for two SARS-CoV-2 spike proteins that contain composite mutations for substitutions were derived from an immunocompromised individual with persistent infection from the time of diagnosis (day 0) through day 154, denoted as day 146* and day 152* (29–31). The day 146* spike protein is derived from hCoV-19/USA/MA-JLL-D146/2020 (EPI_ISL_593557), with WT (Wuhan-Hu-1 SARS-CoV-2 strain) sequences at positions 12 to 18, an amino-terminal domain (NTD) deletion spanning residues 142 to 144, and an additional Y489HRBD mutation, which was observed at an earlier sequencing time point in the immunocompromised host (18, 29, 30). The day 152* spike protein is derived from the hCoV-19/USA/MA-JLL-D152/2020 (EPI_ISL_593558) and contains an additional Y489HRBD mutation. A third construct, RBM-2, was derived from the day 146* spike protein sequence, with two additional substitutions (E484K_RBD_ and K417N_RBD_) as previously described (30). The relevant mutations found in each variant are described in Table 2, and details regarding the lentivirus PN assays are described in the supplemental section. Once produced, PN was performed on serum from mRNA vaccine recipients drawn at the sample collection time points (16, 29). PN titers were reported as the serum dilution required to achieve 50% neutralization (50% inhibitory dilution [ID_50_ value]). The input dilution of serum used was 1:20, which represents the lower limit of quantification for this assay. For ID_50_ analyses, samples that did not achieve 50% neutralization were expressed as 1:10. In cases in which assays on an individual serum sample returned values both >20 and <20, results were assigned a value of 1:15 according to prior methods (31). The pseudovirus neutralization experiment was performed twice with each serum sample, with three replicates included in each experiment.

### Statistical analyses.

Continuous characteristics were described using median and interquartile range (IQR) and compared using Kruskal–Wallis test. Categorical characteristics were described using frequency and percentage and compared using Fisher’s exact test. The correlation between anti-S and PN was evaluated using the robust Theil–Sen linear regression and Spearman correlation. Nonlinearity in trend was evaluated using generalized additive models (GAM). Regression equations are listed in the supplemental material. All analyses were performed in R version 4.1.0 (www.R-project.org).

## References

[1] US FDA. 2021. EUA Authorized Serology Test Performance. Available at: https://www.fda.gov/medical-devices/coronavirus-disease-2019-covid-19-emergency-use-authorizations-medical-devices/eua-authorized-serology-test-performance. Accessed October 5, 2021.

[2] CDC. 2020. Using antibody tests for COVID-19. Available at: https://www.cdc.gov/coronavirus/2019-ncov/lab/resources/antibody-tests.html. Accessed December 11, 2021.

[3] Sherman AC, Desjardins M, Baden LR. 2021. Vaccine-induced SARS-CoV-2 antibody response and the path to accelerating development (determining a correlate of protection). Available at: https://www.sciencedirect.com/science/article/pii/S0272271221000883. Accessed November 24, 2021. 10.1016/j.cll.2021.10.008 PMC8563351 35153045

[4] US FDA. 2021. Spikevax and Moderna COVID-19 Vaccine. Available at: https://www.fda.gov/emergency-preparedness-and-response/coronavirus-disease-2019-covid-19/spikevax-and-moderna-covid-19-vaccine. Accessed March 31, 2021.

[5] Commissioner of the Pfizer-BioNTech COVID-19 Vaccine. FDA. 2021. Available at: https://www.fda.gov/emergency-preparedness-and-response/coronavirus-disease-2019-covid-19/pfizer-biontech-covid-19-vaccine. Accessed March 31, 2021.

[6] US FDA. 2021. Commissioner of the FDA Authorizes Pfizer-BioNTech COVID-19 Vaccine for Emergency Use in Children 5 through 11 Years of Age. Available at: https://www.fda.gov/news-events/press-announcements/fda-authorizes-pfizer-biontech-covid-19-vaccine-emergency-use-children-5-through-11-years-age. Accessed November 22, 2021.

[7] Baden LR, El Sahly HM, Essink B, Kotloff K, Frey S, Novak R, Diemert D, Spector SA, Rouphael N, Creech CB, McGettigan J, Khetan S, Segall N, Solis J, Brosz A, Fierro C, Schwartz H, Neuzil K, Corey L, Gilbert P, Janes H, Follmann D, Marovich M, Mascola J, Polakowski L, Ledgerwood J, Graham BS, Bennett H, Pajon R, Knightly C, Leav B, Deng W, Zhou H, Han S, Ivarsson M, Miller J, Zaks T. 2021. Efficacy and safety of the mRNA-1273 SARS-CoV-2 vaccine. N Engl J Med 384:403–416. doi:10.1056/NEJMoa2035389. 33378609 PMC7787219

[8] Polack FP, Thomas SJ, Kitchin N, Absalon J, Gurtman A, Lockhart S, Perez JL, Pérez Marc G, Moreira ED, Zerbini C, Bailey R, Swanson KA, Roychoudhury S, Koury K, Li P, Kalina WV, Cooper D, Frenck RW, Hammitt LL, Türeci Ö, Nell H, Schaefer A, Ünal S, Tresnan DB, Mather S, Dormitzer PR, Şahin U, Jansen KU, Gruber WC, C4591001 Clinical Trial Group. 2020. Safety and efficacy of the BNT162b2 mRNA COVID-19 vaccine. N Engl J Med 383:2603–2615. doi:10.1056/NEJMoa2034577. 33301246 PMC7745181

[9] US FDA. 2021. Coronavirus (COVID-19) update: FDA expands eligibility for COVID-19 vaccine boosters. Available at: https://www.fda.gov/news-events/press-announcements/coronavirus-covid-19-update-fda-expands-eligibility-covid-19-vaccine-boosters. Accessed December 2, 2021.

[10] Hoffmann M, Kleine-Weber H, Schroeder S, Krüger N, Herrler T, Erichsen S, Schiergens TS, Herrler G, Wu N-H, Nitsche A, Müller MA, Drosten C, Pöhlmann S. 2020. SARS-CoV-2 cell entry depends on ACE2 and TMPRSS2 and is blocked by a clinically proven protease inhibitor. Cell 181:271–280.e8. doi:10.1016/j.cell.2020.02.052. 32142651 PMC7102627

[11] Walls AC, Park Y-J, Tortorici MA, Wall A, McGuire AT, Veesler D. 2020. Structure, function, and antigenicity of the SARS-CoV-2 spike glycoprotein. Cell 181:281–292.e6. doi:10.1016/j.cell.2020.02.058. 32155444 PMC7102599

[12] Rogers TF, Zhao F, Huang D, Beutler N, Burns A, He W-T, Limbo O, Smith C, Song G, Woehl J, Yang L, Abbott RK, Callaghan S, Garcia E, Hurtado J, Parren M, Peng L, Ramirez S, Ricketts J, Ricciardi MJ, Rawlings SA, Wu NC, Yuan M, Smith DM, Nemazee D, Teijaro JR, Voss JE, Wilson IA, Andrabi R, Briney B, Landais E, Sok D, Jardine JG, Burton DR. 2020. Isolation of potent SARS-CoV-2 neutralizing antibodies and protection from disease in a small animal model. Science 369:956–963. doi:10.1126/science.abc7520. 32540903 PMC7299280

[13] Khoury DS, Cromer D, Reynaldi A, Schlub TE, Wheatley AK, Juno JA, Subbarao K, Kent SJ, Triccas JA, Davenport MP. 2021. Neutralizing antibody levels are highly predictive of immune protection from symptomatic SARS-CoV-2 infection. Nat Med 27:1205–1207. doi:10.1038/s41591-021-01377-8. 34002089

[14] McMahan K, Yu J, Mercado NB, Loos C, Tostanoski LH, Chandrashekar A, Liu J, Peter L, Atyeo C, Zhu A, Bondzie EA, Dagotto G, Gebre MS, Jacob-Dolan C, Li Z, Nampanya F, Patel S, Pessaint L, Van Ry A, Blade K, Yalley-Ogunro J, Cabus M, Brown R, Cook A, Teow E, Andersen H, Lewis MG, Lauffenburger DA, Alter G, Barouch DH. 2021. Correlates of protection against SARS-CoV-2 in rhesus macaques. Nature 590:630–634. doi:10.1038/s41586-020-03041-6. 33276369 PMC7906955

[15] Peterhoff D, Glück V, Vogel M, Schuster P, Schütz A, Neubert P, Albert V, Frisch S, Kiessling M, Pervan P, Werner M, Ritter N, Babl L, Deichner M, Hanses F, Lubnow M, Müller T, Lunz D, Hitzenbichler F, Audebert F, Hähnel V, Offner R, Müller M, Schmid S, Burkhardt R, Glück T, Koller M, Niller HH, Graf B, Salzberger B, Wenzel JJ, Jantsch J, Gessner A, Schmidt B, Wagner R. 2021. A highly specific and sensitive serological assay detects SARS-CoV-2 antibody levels in COVID-19 patients that correlate with neutralization. Infection 49:75–82. doi:10.1007/s15010-020-01503-7. 32827125 PMC7441844

[16] Iyer AS, Jones FK, Nodoushani A, Kelly M, Becker M, Slater D, Mills R, Teng E, Kamruzzaman M, Garcia-Beltran WF, Astudillo M, Yang D, Miller TE, Oliver E, Fischinger S, Atyeo C, Iafrate AJ, Calderwood SB, Lauer SA, Yu J, Li Z, Feldman J, Hauser BM, Caradonna TM, Branda JA, Turbett SE, LaRocque RC, Mellon G, Barouch DH, Schmidt AG, Azman AS, Alter G, Ryan ET, Harris JB, Charles RC. 2020. Persistence and decay of human antibody responses to the receptor binding domain of SARS-CoV-2 spike protein in COVID-19 patients. Sci Immunol 5. doi:10.1126/sciimmunol.abe0367. PMC7857394 33033172

[17] Wajnberg A, Amanat F, Firpo A, Altman DR, Bailey MJ, Mansour M, McMahon M, Meade P, Mendu DR, Muellers K, Stadlbauer D, Stone K, Strohmeier S, Simon V, Aberg J, Reich DL, Krammer F, Cordon-Cardo C. 2020. Robust neutralizing antibodies to SARS-CoV-2 infection persist for months. Science 370:1227–1230. doi:10.1126/science.abd7728. 33115920 PMC7810037

[18] Choi B, Choudhary MC, Regan J, Sparks JA, Padera RF, Qiu X, Solomon IH, Kuo H-H, Boucau J, Bowman K, Adhikari UD, Winkler ML, Mueller AA, Hsu TY-T, Desjardins M, Baden LR, Chan BT, Walker BD, Lichterfeld M, Brigl M, Kwon DS, Kanjilal S, Richardson ET, Jonsson AH, Alter G, Barczak AK, Hanage WP, Yu XG, Gaiha GD, Seaman MS, Cernadas M, Li JZ. 2020. Persistence and evolution of SARS-CoV-2 in an immunocompromised host. N Engl J Med 383:2291–2293. doi:10.1056/NEJMc2031364. 33176080 PMC7673303

[19] Yurkovetskiy L, Wang X, Pascal KE, Tomkins-Tinch C, Nyalile TP, Wang Y, Baum A, Diehl WE, Dauphin A, Carbone C, Veinotte K, Egri SB, Schaffner SF, Lemieux JE, Munro JB, Rafique A, Barve A, Sabeti PC, Kyratsous CA, Dudkina NV, Shen K, Luban J. 2020. Structural and functional analysis of the D614G SARS-CoV-2 spike protein variant. Cell 183:739–751.e8. doi:10.1016/j.cell.2020.09.032. 32991842 PMC7492024

[20] Goldberg Y, Mandel M, Bar-On YM, Bodenheimer O, Freedman L, Haas EJ, Milo R, Alroy-Preis S, Ash N, Huppert A. 2021. Waning immunity after the BNT162b2 vaccine in Israel. N Engl J Med 385:e85. doi:10.1056/NEJMoa2114228. 34706170 PMC8609604

[21] Pegu A, O’Connell S, Schmidt SD, O’Dell S, Talana CA, Lai L, Albert J, Anderson E, Bennett H, Corbett KS, Flach B, Jackson L, Leav B, Ledgerwood JE, Luke CJ, Makowski M, Nason MC, Roberts PC, Roederer M, Rebolledo PA, Rostad CA, Rouphael NG, Shi W, Wang L, Widge AT, Yang ES, Group§ T, mRNA-1273 S, Beigel JH, Graham BS, Mascola JR, Suthar MS, McDermott AB, Doria-Rose NA, Arega J, Beigel JH, Buchanan W, Elsafy M, Hoang B, Lampley R, Kolhekar A, Koo H, Luke C, Makhene M, Nayak S, Pikaart-Tautges R, Roberts PC, Russell J, Sindall E, Albert J, Kunwar P, Makowski M, Anderson EJ, Bechnak A, Bower M, Camacho-Gonzalez AF, Collins M, Drobeniuc A, Edara VV, Edupuganti S, Floyd K, Gibson T, Ackerley CMG, Johnson B, Kamidani S, Kao C, Kelley C, Lai L, Macenczak H, McCullough MP, Peters E, Phadke VK, Rebolledo PA, Rostad CA, Rouphael N, Scherer E, Sherman A, Stephens K, Suthar MS, Teherani M, Traenkner J, Winston J, Yildirim I, Barr L, Benoit J, Carste B, Choe J, Dunstan M, Erolin R, ffitch J, Fields C, Jackson LA, Kiniry E, Lasicka S, Lee S, Nguyen M, Pimienta S, Suyehira J, Witte M, Bennett H, Altaras NE, Carfi A, Hurley M, Leav B, Pajon R, Sun W, Zaks T, Coler RN, Larsen SE, Neuzil KM, Lindesmith LC, Martinez DR, Munt J, Mallory M, Edwards C, Baric RS, Berkowitz NM, Boritz EA, Carlton K, Corbett KS, Costner P, Creanga A, Doria-Rose NA, Douek DC, Flach B, Gaudinski M, Gordon I, Graham BS, Holman L, Ledgerwood JE, Leung K, Lin BC, Louder MK, Mascola JR, McDermott AB, Morabito KM, Novik L, O’Connell S, O’Dell S, Padilla M, Pegu A, Schmidt SD, Shi W, Swanson PA, Talana CA, Wang L, Widge AT, Yang ES, Zhang Y, Chappell JD, Denison MR, Hughes T, Lu X, Pruijssers AJ, Stevens LJ, Posavad CM, Gale M Jr, Menachery V, Shi P-Y, et al. 2021. Durability of mRNA-1273 vaccine–induced antibodies against SARS-CoV-2 variants. Science. Available at: https://www.science.org/doi/abs/10.1126/science.abj4176. Accessed September 8, 2021. 10.1126/science.abj4176 PMC8691522 34385356

[22] Steensels D, Pierlet N, Penders J, Mesotten D, Heylen L. 2021. Comparison of SARS-CoV-2 Antibody Response Following Vaccination With BNT162b2 and mRNA-1273. JAMA 326:1533. doi:10.1001/jama.2021.15125. 34459863 PMC8406205

[23] Röltgen K, Nielsen SCA, Arunachalam PS, Yang F, Hoh RA, Wirz OF, Lee AS, Gao F, Mallajosyula V, Li C, Haraguchi E, Shoura MJ, Wilbur JL, Wohlstadter JN, Davis MM, Pinsky BA, Sigal GB, Pulendran B, Nadeau KC, Boyd SD et al. 2021. mRNA vaccination compared to infection elicits an IgG-predominant response with greater SARS-CoV-2 specificity and similar decrease in variant spike recognition. Available at: https://www.medrxiv.org/content/10.1101/2021.04.05.21254952v1. Accessed February 10, 2022.

[24] Wheeler SE, Shurin GV, Yost M, Anderson A, Pinto L, Wells A, Shurin MR. 2021. Differential antibody response to mRNA COVID-19 vaccines in healthy subjects. Microbiol Spectr 9. doi:10.1128/Spectrum.00341-21. PMC8552678 34346750

[25] El Sahly HM, Baden LR, Essink B, Doblecki-Lewis S, Martin JM, Anderson EJ, Campbell TB, Clark J, Jackson LA, Fichtenbaum CJ, Zervos M, Rankin B, Eder F, Feldman G, Kennelly C, Han-Conrad L, Levin M, Neuzil KM, Corey L, Gilbert P, Janes H, Follmann D, Marovich M, Polakowski L, Mascola JR, Ledgerwood JE, Graham BS, August A, Clouting H, Deng W, Han S, Leav B, Manzo D, Pajon R, Schödel F, Tomassini JE, Zhou H, Miller J, COVE Study Group. 2021. Efficacy of the mRNA-1273 SARS-CoV-2 vaccine at completion of blinded phase. N Engl J Med 385:1774–1785. doi:10.1056/NEJMoa2113017. 34551225 PMC8482810

[26] FDA. 2021. Convalescent plasma EUA letter of authorization 06032021. Available at: https://www.fda.gov/media/141477/download. Accessed October 8, 2021.

[27] Roche Diagnostics. 2020. Elecsys anti-SARS-CoV-2. Available at: https://www.fda.gov/media/137605/download. Accessed June 23, 2021.

[28] Riester E, Findeisen P, Hegel JK, Kabesch M, Ambrosch A, Rank CM, Pessl F, Laengin T, Niederhauser C. 2021. Performance evaluation of the Roche Elecsys Anti-SARS-CoV-2 S immunoassay. J Virol Methods 297:114271. doi:10.1016/j.jviromet.2021.114271. 34461153 PMC8393518

[29] Clark SA, Clark LE, Pan J, Coscia A, McKay LGA, Shankar S, Johnson RI, Brusic V, Choudhary MC, Regan J, Li JZ, Griffiths A, Abraham J. 2021. SARS-CoV-2 evolution in an immunocompromised host reveals shared neutralization escape mechanisms. Cell 184:2605–2617.e18. doi:10.1016/j.cell.2021.03.027. 33831372 PMC7962548

[30] Nabel KG, Clark SA, Shankar S, Pan J, Clark LE, Yang P, Coscia A, McKay LGA, Varnum HH, Brusic V, Tolan NV, Zhou G, Desjardins M, Turbett SE, Kanjilal S, Sherman AC, Dighe A, LaRocque RC, Ryan ET, Tylek C, Cohen-Solal JF, Darcy AT, Tavella D, Clabbers A, Fan Y, Griffiths A, Correia IR, Seagal J, Baden LR, Charles RC, Abraham J. 2022. Structural basis for continued antibody evasion by the SARS-CoV-2 receptor binding domain. Science 375:eabl6251. doi:10.1126/science.abl6251. 34855508 PMC9127715

[31] Jackson LA, Anderson EJ, Rouphael NG, Roberts PC, Makhene M, Coler RN, McCullough MP, Chappell JD, Denison MR, Stevens LJ, Pruijssers AJ, McDermott A, Flach B, Doria-Rose NA, Corbett KS, Morabito KM, O'Dell S, Schmidt SD, Swanson PA, Padilla M, Mascola JR, Neuzil KM, Bennett H, Sun W, Peters E, Makowski M, Albert J, Cross K, Buchanan W, Pikaart-Tautges R, Ledgerwood JE, Graham BS, Beigel JH, mRNA-1273 Study Group. 2020. An mRNA vaccine against SARS-CoV-2—preliminary report. N Engl J Med 383:1920–1931. doi:10.1056/NEJMoa2022483. 32663912 PMC7377258

