# Supplementary material for: The Effect of Vaccine Type and SARS-CoV-2 Lineage on Commercial SARS-CoV-2 Serologic and Pseudotype Neutralization Assays in mRNA Vaccine Recipients
Source: Microbiol Spectr. 2022 Mar 21;10(2):e00211-22. doi: 10.1128/spectrum.00211-22 (PMC9045317; doi:10.1128/spectrum.00211-22)
Supplement: SUPPLEMENTAL FILE 1 — Supplemental material. Download SPECTRUM00211-22_Supp_1_seq6.pdf, PDF file, 1.0 MB [file spectrum00211-22_supp_1_seq6.pdf]

Supplemental Material

Methods

Lentivirus pseudotype production

Human codon-optimized SARS-CoV-2 (GenBank ID: QJA16508.1 residues 1-1246) S protein was cloned into the pCAGGS expression vector and modified to include HIV gp41 residues (NRVRQGYS) as previously described [1], replacing C-terminal residues 1247–1273. The SARS-CoV-2 spike constructs for D614G S wild-type (WT), Alpha, Beta, day 146*, day 152*, and RBM-2 pseudotypes were previously described [1,2]. Spike protein lentivirus pseudotypes were packaged in HEK293T cells, harvested, filtered, concentrated, and stored at -80°C, as previously described [1,2].

Pseudotype neutralization assays

SARS-CoV-2 WT or variant lentivirus pseudotypes were pre-incubated with serial dilutions of polyclonal human sera in the presence of DMEM supplemented with 0.5 µg ml^{-1} of polybrene for 1 hour at 37°C. We added pseudotype-serum mixtures to monolayers of HEK293T cells overexpressing ACE2 for 24 hours at 37°C. We then replaced the media with DMEM supplemented with 10% (v/v) FBS, 1% (v/v) penicillin-streptomycin, and 25 mM HEPES. We performed flow cytometry 48 hours after initial infection with an iQue Screener PLUS (Intellicyt) to quantify the percent of GFP positive cells. We calculated percent relative entry and percent neutralization using the following equations: (1) Relative entry (%) = (%GFP-positive cells in the presence of antibody/%GFP in the absence of antibody) x 100%; (2) Neutralization (%) = [1 - (%GFP-positive cells in the presence of antibody/%GFP in the absence of antibody)] x 100%. We generated response curves with a 4-parameter nonlinear function and reported titers as the antibody concentration required to achieve 50% inhibitory dilution (ID_{50} value) for serum in GraphPad Prism. Of note, some of the participant samples analyzed for ID50 were also used for a prior investigation for a different type of analysis [2].

References

1. Clark SA, Clark LE, Pan J, et al. SARS-CoV-2 evolution in an immunocompromised host reveals shared neutralization escape mechanisms. Cell **2021**; 184:2605-2617.e18.

2. Nabel KG, Clark SA, Shankar S, et al. Structural basis for continued antibody evasion by the SARS-CoV-2 receptor binding domain. Science 0:eabl6251.

<u>Regression Equations</u>

Regression equations correspond to the solid lines in Figure 3 and 4, where s(log10(Anti-S),1) and s(log10(Anti-S),2) are respectively the 1st and 2nd smoothing basis functions used in the generalized additive model.

BNT162b2 WT samples:
log10(ID50) = 2.11- 0.10*s(log10(Anti-S),1) + 0.64*s(log10(Anti-S),2) + random-error
mRNA-1273 WT samples:
log10(ID50) = 2.00 - 0.38*s(log10(Anti-S),1) + 0.73*s(log10(Anti-S),2) + random-error

BNT162b2 Alpha (B.1.1.7) samples:
log10(ID50) = 1.89 - 0.30*s(log10(Anti-S),1) + 0.58*s(log10(Anti-S),2) + random-error
mRNA-1273 Alpha (B.1.1.7) samples:
log10(ID50) = 1.84 - 0.47*s(log10(Anti-S),1) + *s(log10(Anti-S),2) + random-error

BNT162b2 Beta (B.1.3.5.1) samples:
log10(ID50) =1.66 - 0.43*s(log10(Anti-S),1) + 0.48*s(log10(Anti-S),2) + random-error
mRNA-1273 Beta (B.1.3.5.1) samples:
log10(ID50) =1.58 - 0.73*s(log10(Anti-S),1) + 0.55*s(log10(Anti-S),2) + random-error

BNT162b2 Gamma (P.1) samples:
log10(ID50) = 1.58 - 0.54*s(log10(Anti-S),1) + 0.47*s(log10(Anti-S),2) + random-error
mRNA-1273 Gamma (P.1) samples:
log10(ID50) = 1.52 - 0.91*s(log10(Anti-S),1) + 0.57*s(log10(Anti-S),2) + random-error

BNT162b2 Day 146 samples:
log10(ID50) = 1.82 - 0.22*s(log10(Anti-S),1) + 0.49*s(log10(Anti-S),2) + random-error
mRNA-1273 Day 146 samples:
log10(ID50) = 1.69 - 0.79*s(log10(Anti-S),1) + 0.65*s(log10(Anti-S),2) + random-error

BNT162b2 Day 152 samples:
log10(ID50) = 1.63 - 0.10*s(log10(Anti-S),1) + 0.37*s(log10(Anti-S),2) + random-error
mRNA-1273 Day 152 samples:
log10(ID50) = 1.57 - 0.42*s(log10(Anti-S),1) + 0.48*s(log10(Anti-S),2) + random-error

BNT162b2 RBM2 samples:
log10(ID50) = 1.79 - 0.06*s(log10(Anti-S),1) + 0.39*s(log10(Anti-S),2) + random-error
mRNA-1273 RBM2 samples:
log10(ID50) = 1.75 - 0.74*s(log10(Anti-S),1) + 0.58*s(log10(Anti-S),2) + random-error

Supplemental Table 1. Convalescent Plasma Donor Demographics

	**Convalescent Plasma Donors , n=66**
**Median Age, years (IQR)**	56.5 (48.5-64.7
**Female Sex - no. (%)**	36 (54.5)
**Race - no (%)**	
White	43 (65.2)
Black	11 (16.7)
Asian	6 (9.1)
Native American/Alaskan Native+A10	0 (0.0)
Other	6 (9.1)
**Ethnicity - no. (%)**	
Hispanic	5 (7.6)
Non-Hispanic	59 (89.4)
Not available	2 (3.0)
**COVID-19 Disease Severity - no. (%)**	
Ambulatory mild disease (WHO score 2-3)	32 (48.5)
Hospitalized, moderate disease (WHO score 4-5)	28 (42.4)
Hospitalized, severe disease (WHO score 6-9)	6 (9.1)
**Median days from symptoms onset to screening (IQR)**	74 (61-105)
**Median days from positive PCR to onset to screening (IQR)**	70 (55-100)

IQR=Interquartile range; PCR=polymerase chain reaction

Supplemental Table 2. Pseudotype neutralizing antibody levels (ID_{50}) at 49 to 56 days after the first vaccine dose (28 days after the second vaccine dose) by SARS-CoV-2 strain and vaccine type, median (IQR).

	**mRNA-1273**	**BNT162b2**	p-values
**WT**	654.4 (564.8-995.5)	394.3 (280.0 - 623.9)	0.027
**Alpha**	416.5 (338.9 - 498.7)	205.9 (130.7 - 322.3)	0.008
**Beta**	186.1 (147.6 - 247.5)	108.6 (77.6 - 134.8)	0.004
**Gamma**	165.8 (122.0 - 228.7)	72.1 (48.0 -114.8)	0.002
**Day 146***	329.2 (197.1- 426.0)	126.1 (93.9 - 139.8)	0.009
**Day 152***	131.9 (96.1 - 211.4)	76.5 (60.4 - 96.6)	0.003
**RBM-2**	171.3 (127.0 - 212.7)	104.9 (93.9 - 135.6)	0.042

Supplemental Figure 1. Measurement of A) IgM, B) IgA, and C) IgG isotype levels (µg/mL) to the SARS-CoV-2 S protein RBD in mRNA-1273 (*n* = 12) and BNT162b2 (*n* = 8) vaccine recipients. Each dot represents a unique measurement of isotype levels; best-fit lines (LOWESS fit) are shown with corresponding 95% CI colored according to vaccine type.

A

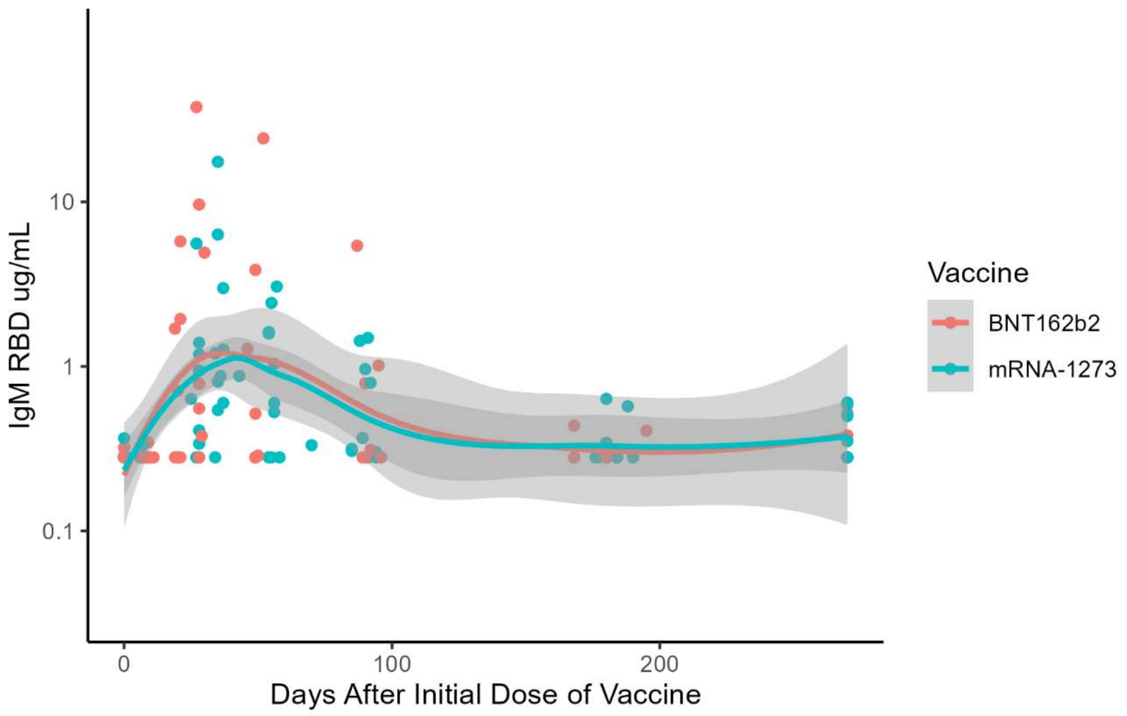
IgM RBD ug/mL
10
1
0.1
0
100
200
Days After Initial Dose of Vaccine
Vaccine
BNT162b2
mRNA-1273

B

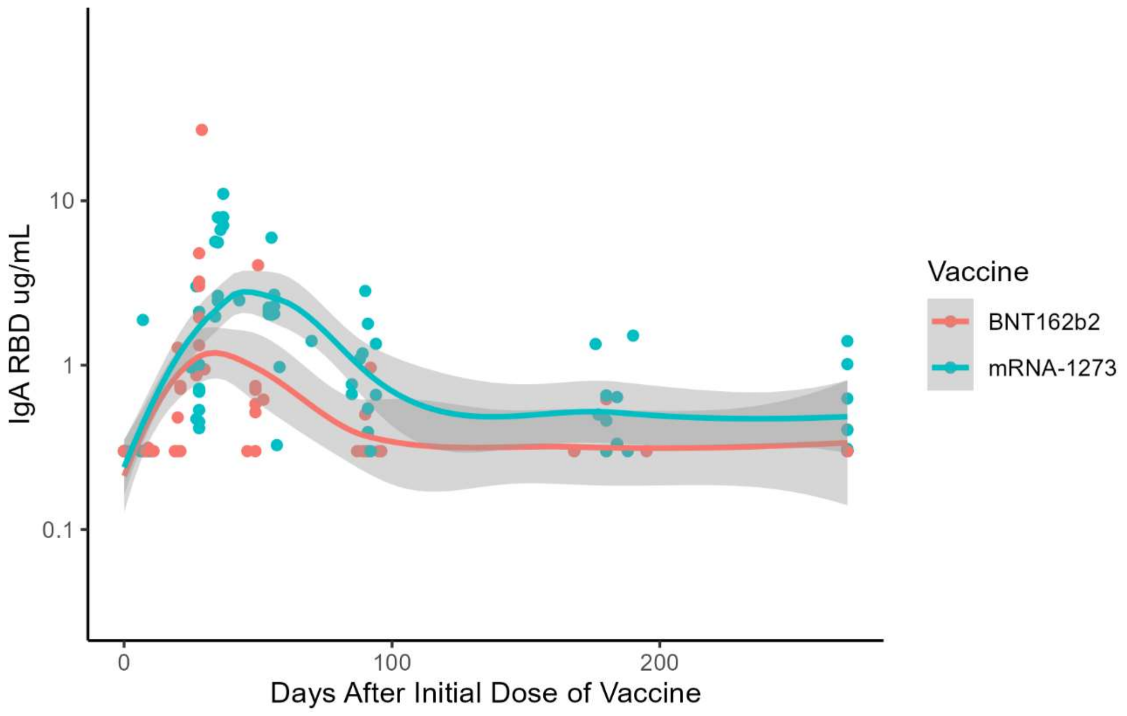
IgA RBD ug/mL
10
1
0.1
0
100
200
Days After Initial Dose of Vaccine
Vaccine
BNT162b2
mRNA-1273

C

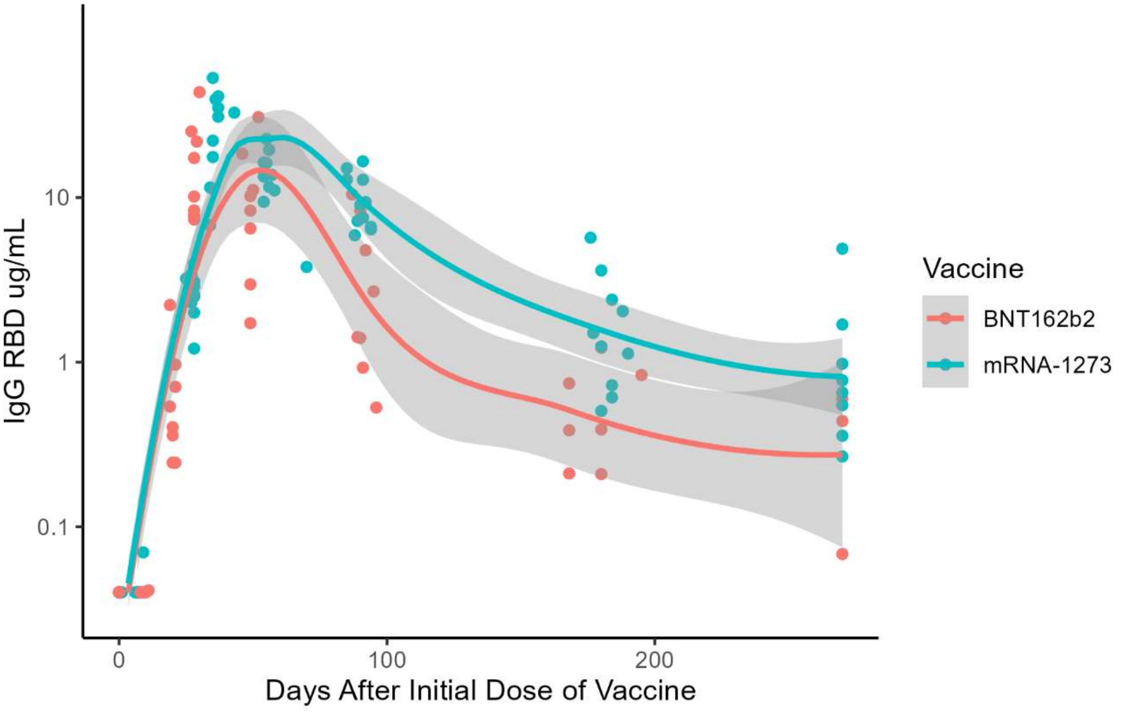

IgG RBD ug/mL
10
1
0.1
0
100
200
Days After Initial Dose of Vaccine
Vaccine
BNT162b2
mRNA-1273
